# Detection of East/Central/South African Genotype of Chikungunya Virus in Myanmar, 2010

**DOI:** 10.3201/eid2008.131431

**Published:** 2014-08

**Authors:** Mya Myat Ngwe Tun, Kyaw Zin Thant, Shingo Inoue, Takeshi Nabeshima, Kotaro Aoki, Aung Kyaw Kyaw, Tin Myint, Thi Tar, Kay Thwe Thwe Maung, Daisuke Hayasaka, Kouichi Morita

**Affiliations:** Nagasaki University, Nagasaki, Japan (M.M. Ngwe Tun, S. Inoue, T. Nabeshima, K. Aoki, A.K. Kyaw, D. Hayasaka, K. Morita);; Upper Myanmar Medical Research, Pyin Oo Lwin, Myanmar (K.Z. Thant, A.K. Kyaw, K.T.T. Maung);; Mandalay Children Hospital, Mandalay, Myanmar (T. Myint, T. Tar)

**Keywords:** chikungunya virus, molecular epidemiology, Myanmar, viruses, genotype East Central South African genotype, genotypes

## Abstract

In 2010, chikungunya virus of the East Central South African genotype was isolated from 4 children in Myanmyar who had dengue-like symptoms. Phylogenetic analysis of the E1 gene revealed that the isolates were closely related to isolates from China, Thailand, and Malaysia that harbor the A226V mutation in this gene.

Chikungunya virus (CHIKV; family *Togaviridae,* genus *Alphavirus*) is transmitted to humans by the bite of infected mosquitoes of the genus *Aedes*, particularly *Ae. aegypti* and *Ae. albopictus* (*1*). Dengue viruses (DENVs; family *Flaviviridae*, genus *Flavivirus*) are transmitted by the same mosquito vectors. CHIKV and DENV are often found co-circulating (*2*,*3*) and cause similar clinical signs and symptoms, including fever, arthralgia, headache, rash, and, in some cases, hemorrhage. Hemorrhage, however, is rare in CHIKV infection. Because of the similarity in signs and symptoms, misdiagnosis and underreporting of chikungunya infection in dengue-endemic areas are common (*3*). 

Chikungunya is endemic to the tropical areas of Africa and Asia (*5*) and generally manifests as mild illness, but outbreaks in the Indian Ocean region have shown that the disease can lead to severe and life-threatening complications (*4*). CHIKV strains are clustered into 3 distinct genotypes: West African, East/Central/South African (ECSA), and Asian (*5*). Reemergence of CHIKV of the ECSA genotype began in Kenya in 2004, and during 2005–2010, the genotype spread to Comoros, Madagascar, Reunion Island, India, Sri Lanka, Singapore, Thailand, Malaysia, China, and Italy (*6*).

In Myanmar, CHIKV infection was serologically confirmed in 1973, and the virus was indicated to be of the Asian genotype (no sequence data available) (*5*,*7*). A few serologic studies in this country have found CHIKV-specific antibodies in patients who were suspected of having dengue virus infection (*7*–*9*), but no reports have been published on the molecular epidemiology of CHIKV. We conducted a serologic and molecular epidemiologic surveillance study of possible CHIKV infection among children who had a clinical diagnosis of dengue fever in Myanmar during 2010.

## The Study

**Figure 1:**
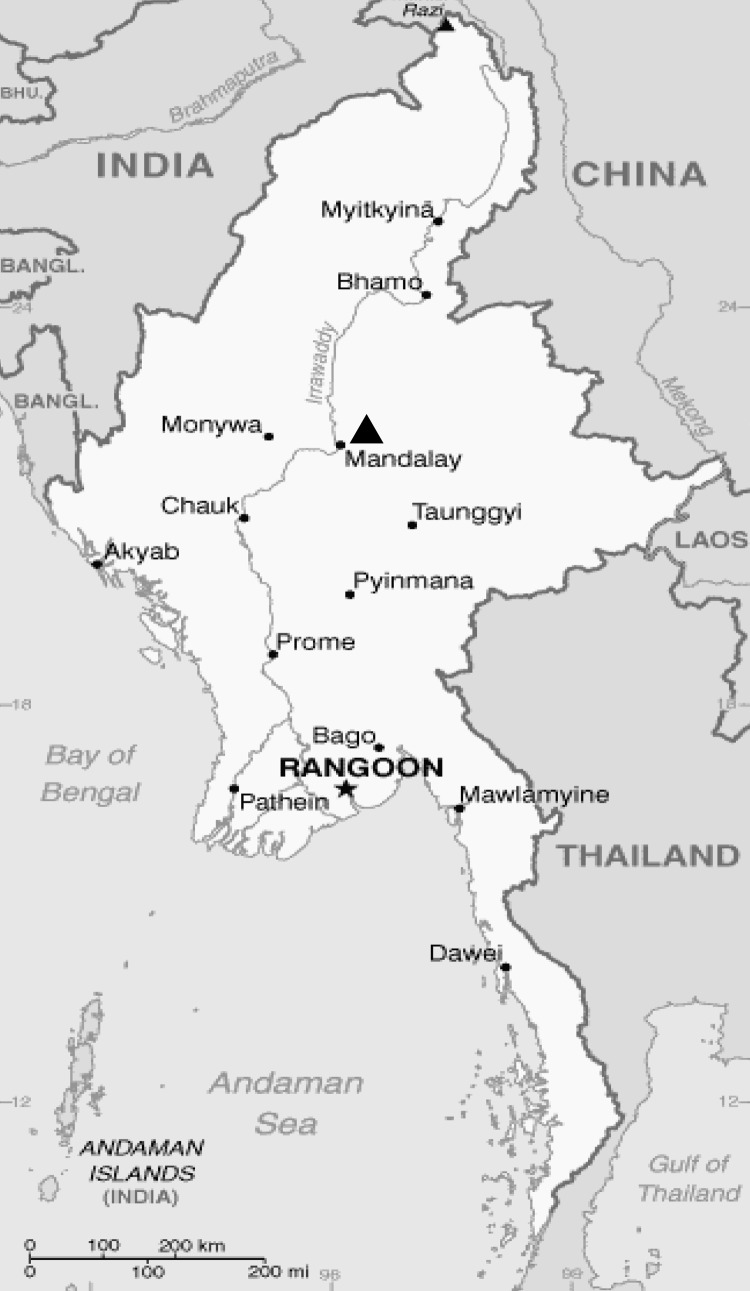
Location of Mandalay Children Hospital (triangle) in Myanmar.

During July–October 2010, serum samples were collected from 116 children (<12 years of age) who were admitted to Mandalay Children Hospital in Mandalay, Myanmar (Figure 1). These children had received a clinical diagnosis of dengue, signs and symptoms of which included fever, hemorrhage, shock, and thrombocytopenia. A blood sample was collected from each child, 107 during the acute phase of illness (1–7 days after onset of fever) and 9 during the convalescent phase (8–16 days after onset of fever). Serum samples were stored at −70°C until further analysis. Informed consent was obtained from the accompanying parents or legal guardians before blood samples were collected by following the research protocol (approval no. 1/2010 by the Institutional Ethical Committee on Medical Research Involving Human Subjects in Myanmar). 

All 116 serum samples were tested for IgM against CHIKV and DENV by using in-house IgM capture ELISAs and for IgG against CHIKV and flaviviruses by using in-house IgG indirect ELISAs (*10*). Results of IgM capture ELISAs showed 6 (5.2%) samples were positive for IgM against CHIKV only, 47 (40.5%) for IgM against DENV only, and 7 (6.0%) for IgM against CHIKV and DENV (Table 1). Results of IgG indirect ELISA showed 8 (6.9%) samples were positive for IgG against CHIKV only, 47 (40.5%) for IgG against flavivirus only, and 9 (7.8%) for IgG against CHIKV and flavivirus (Table 1).

**Table 1:** Antibody profiles of samples from 116 children who had suspected dengue virus infection, Myanmar, 2010*

Profile	CHIKV		DENV IgM	Flavivirus IgG	Total no. patients
	IgM	IgG			
A	+	+	–	+	2
B	+	+	–	–	4
C	+	–	–	+	0
D	+	–	–	–	0
E	–	+	+	+	4
F	–	+	+	–	2
G	–	–	+	+	32
H	–	–	+	–	9
I	+	+	+	+	3
J	+	+	+	–	0
K	+	–	+	+	2
L	+	–	+	–	2
M	–	+	–	+	0
N	–	+	–	–	2
O	–	–	–	+	13
P	–	–	–	–	41

*CHIKV, chikungunya virus; DENV, dengue virus; +, positive; –, negative.

For PCR testing, 10 μL of serum from each acute-phase sample was inoculated onto *Ae. albopictus* clone C6/36 mosquito cells, which were then incubated at 28°C for 7 days. RNA was extracted from infected culture fluid by using a QIAamp Viral RNA Mini Kit (QIAGEN, Hilden, Germany), and reverse transcription PCR was performed by using Superscript III Reverse Transcriptase (Invitrogen, Carlsbad, CA, USA). PCR was conducted by using Takara Tks Gflex DNA polymerase (Takara Bio, Shiga, Japan). All kits were used according to the manufacturer’s instructions.

**Figure 2:**
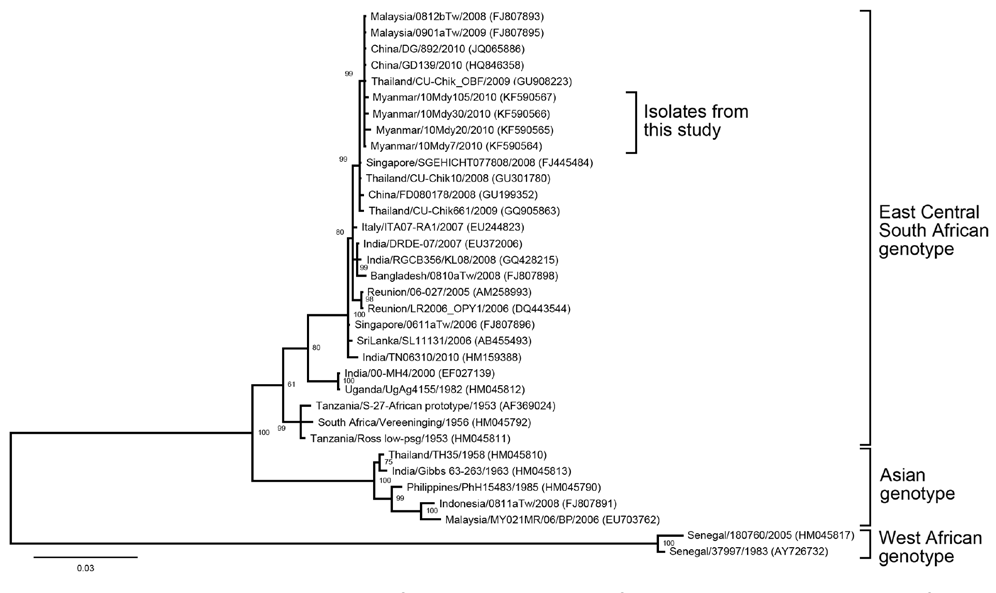
Phylogenetic tree constructed on the basis of whole E1 gene sequences of chikungunya viruses, showing location of 4 isolates obtained from children in Myanmar within the East Central South African genotype. Representative strains of each genotype obtained from GenBank are named by country of origin, strain name, year of isolation, and accession number (in parentheses). Bootstrap values are indicated at branch nodes. Scale bar indicates nucleotide substitutions per site.

For the 107 acute-phase serum samples tested, 4 CHIKV strains were isolated from infected culture fluid. Detailed information about the children from which these samples were collected and the test results are shown in Table 2. The complete E1 gene nucleotide sequences (positions 9952–11271) for the 4 CHIKV isolates were determined and deposited into GenBank (accession nos. KF590564–7), and nucleotide sequences were aligned by using MAFFT version 7.058b (http://mafft.cbrc.jp/alignment/software). A Bayesian maximum clade credibility tree was constructed by using MrBayes version 3.1.2 (http://mrbayes.sourceforge.net), and a phylogenetic tree was drawn by using FigTree version 1.4.0 software (http://beast.bio.ed.ac.uk/FigTree). The phylogenetic tree of CHIKV was inferred for the 4 strains from Myanmar and 30 reference strains available from GenBank; results showed that the 4 CHIKV isolates from Myanmar belonged to the ECSA genotype (Figure 2). 

**Table 2:** Characteristics of and testing results for 4 patients who had suspected dengue virus infection and from whom chikungunya virus was isolated, Myanmar. 2010*

Patient no.	Age, y	Days from illness onset to blood extraction*	Diagnosis at admission	CHIKV				DENV		Flavivirus IgG
				IgM	IgG	RT-PCR		IgM	RT-PCR	
7	6.5	1	DHF I	Neg	Neg	Pos		Pos	Neg	Pos
20	11	2	DHF II	Neg	Neg	Pos		Neg	Neg	Neg
30	6	4	DF	Neg	Neg	Pos		Neg	Neg	Neg
105	7	3	DHF II	Neg	Neg	Pos		Neg	Neg	Pos

*DHF grades I and II classified according to World Health Organization criteria, 1997. CHIKV, chikungunya virus; DENV, dengue virus; RT-PCR, reverse transcription PCR; DHF, dengue hemorrhagic fever; Pos, positive; Neg, Negative; DF, dengue fever.

## Conclusions

Serologic results from this study confirmed that 47 (40.5%) of the 116 children who had received a clinical diagnosis of dengue had DENV infection only, but 6 (5.2%) had IgM capture ELISA results showing infection with CHIKV; CHIKV was isolated from 4 of these patients. Cross-reactivity in the IgM response against other alphaviruses in the same antigenic complex to which CHIKV belongs is possible (*11*), but other alphaviruses have not been reported in Myanmar. In addition, 7 (6.0%) of the children had positive test results for IgM against CHIKV and DENV. This finding could be the result of sequential infection by the 2 viruses in a short period or by concurrent infection with both viruses. Concurrent CHIKV and DENV infections have been reported in Gabon and Singapore (*3*,*12*). A total of 6.9%, 40.5%, and 7.8% of children tested showed positive results for IgG against CHIKV, flavivirus, and CHIKV/flavivirus, respectively. This result suggests that arbovirus infections are common in Myanmar.

The 4 CHIKV isolates that we obtained belonged to an ECSA genotype subgroup that has the A226V mutation in the E1 gene. One of the 4 isolates (Myanmar/10Mdy20/2010) had an additional amino acid change (E1-E209V). All isolates had 99% nucleotide sequence similarity to isolates from China, Thailand, and Malaysia. Outbreaks caused by ECSA genotype CHIKV were reported in Malaysia in 2008–2009, in Thailand in 2008–2009, and in China in 2010 (*7*,*13*,*14*). The timing of these findings suggests that the strains of CHIKV we found in Myanmar were introduced from neighboring countries. Human migratory movements between the borders of Myanmar and neighboring countries such as India, Thailand, and China for trade and travel purposes are increasing and could be a contributing factor to the movement of the virus and the outbreaks. In Southeast Asia, CHIKV strains have been isolated not only from humans and mosquitoes but also from monkeys (*15*). We recommend surveillance for chikungunya and its vectors to prevent future outbreaks of CHIKV infection among the human population in Myanmar.
